# Number of Teeth, Oral Self-care, Eating Speed, and Metabolic Syndrome in an Aged Japanese Population

**DOI:** 10.2188/jea.JE20170210

**Published:** 2019-01-05

**Authors:** Mizuki Saito, Yoshihiro Shimazaki, Toshiya Nonoyama, Yasushi Tadokoro

**Affiliations:** 1 Department of Preventive Dentistry and Dental Public Health, School of Dentistry, Aichi Gakuin University, Nagoya, Aichi, Japan; 2 Mie Dental Association, Tsu, Mie, Japan

**Keywords:** dental public health, eating behavior, oral hygiene, geriatric dentistry, epidemiology

## Abstract

**Background:**

Many studies have reported that oral health status is associated with various systemic health issues. This study examined the correlations among oral health, lifestyle factors, and metabolic syndrome (MetS) in aged participants.

**Methods:**

We analyzed cross-sectional oral and medical health checkup data from 2,379 participants aged 75 and 80 years. MetS was diagnosed according to the Harmonization criteria, with the exception of the criterion for central obesity, and body mass index was used instead of waist circumference. Logistic regression analyses were performed to evaluate the correlation between oral health status and lifestyle factors and MetS in both sexes and by sex.

**Results:**

In both sexes, the odds ratio (OR) for MetS was 1.54 (95% confidence interval [CI], 1.10–2.17) among those who had 0–9 teeth compared with those with 20–28 teeth. MetS was significantly more likely for those eating quickly than those eating slowly (OR 2.06; 95% CI, 1.35–3.16). Participants using secondary oral hygiene products every day had a significantly lower OR (0.71; 95% CI, 0.55–0.92) for MetS than did those who did not. Participants with 0–9 teeth who ate quickly had a significantly higher OR (2.48; 95% CI, 1.06–5.78) for MetS compared with those with 20–28 teeth who ate slowly.

**Conclusion:**

These results suggest that maintaining teeth, eating slowly, and using secondary oral hygiene products every day are associated with a lower likelihood of MetS in the aged population.

## INTRODUCTION

Metabolic syndrome (MetS) is a combination of risk factors for coronary artery disease (CAD) and type 2 diabetes mellitus, including high blood pressure, hyperglycemia, abdominal obesity, hypertriglyceridemia, and a low level of high density lipoprotein (HDL) cholesterol. The simultaneous occurrence of these risk factors significantly increases the risk of CAD and diabetes.^1^ Thus, MetS has attracted attention as an important health concern.

MetS is affected by diet and exercise habits.^2^ Many studies have also reported a relationship between MetS and oral health status, such as dental caries, periodontal disease, and the number of remaining teeth.^3^^–^^6^ This may be because tooth loss caused by dental caries and periodontal disease affects masticatory function and diet,^7^^,^^8^ and chronic inflammation and oxidative stress are common risk factors among individuals with periodontal disease and MetS.^9^

Most previous studies on this participant have examined the relationship between oral health status and MetS mainly in middle-aged populations,^3^^–^^6^ but few have been conducted in aged populations. According to the 2014 National Health and Nutrition Survey in Japan, the prevalence of MetS in the Japanese adult population is increasing with advancing age, and the percentage of individuals with MetS ≥75 years is approximately 25%, which is higher than that in middle-aged and younger populations. MetS poses significant risks of dementia and cognitive decline, as well as CAD and diabetes, in elderly individuals.^10^^,^^11^ MetS is also an independent risk factor for progressive disability and decreased mobility in the elderly.^12^^,^^13^ Therefore, MetS is an important health problem associated with mental and physical depression and the need for care in elderly individuals.

Clarifying the factors associated with MetS is important for preventing MetS in the elderly population. This study examined the correlations among oral health including the number of remaining teeth, lifestyle factors, and MetS in the aged population. We also examined the correlation between a combination of the number of teeth and eating speed with MetS.

## METHODS

### Study participants

The participants of this study were insured by the public healthcare insurance system for the elderly aged ≥75 years in Japan. Such individuals can receive a general health examination once annually. Since 2014, the government has subsidized oral health examinations for the aged population to each prefectural insurer, called “wide area unions for the late-stage medical care system”, and the Mie Prefecture insurer covers oral health examinations for those aged 75 and 80 years. This cross-sectional study was performed using existing records obtained from general and oral health examinations covered by the insurer for elderly people aged ≥75 years in Mie Prefecture.

The insurer for the aged population in Mie Prefecture sent an oral health examination ticket to 75- and 80-year-old individuals in September 2014. The oral examinations were performed at a registered Mie Dental Association Clinic from October 1, 2014 to November 30, 2014. The general health examinations were performed at a registered medical clinic from July 1, 2014 to November 30, 2014. There were 17,338 people aged 75 years and 16,040 people aged 80 years in Mie Prefecture in 2014. Totals of 7,995 (46.1%) and 6,699 (41.8%) 75- and 80-year-old individuals received general health examinations, respectively, and 2,865 (16.5%) and 2,119 (13.2%) received oral health examinations, respectively. Individuals who had current or previous medical histories of cardiovascular disease (CVD) or renal insufficiency were excluded from the analyses. We analyzed the data of 2,379 individuals, including 1,469 participants aged 75 years and 910 participants aged 80 years who received both general and oral heath examinations and who met the requirements for the analyses. The health examinations were conducted not for the survey, but rather for periodic health checkups for the aged population, so we did not obtain consent from the participants. We obtained permission from the insurer to use the de-identified data for this study, and this study was approved by the Institutional Review Board (IRB) of Aichi Gakuin University, School of Dentistry (approval number 443) and was conducted in full accordance with the World Medical Association Declaration of Helsinki. This study conforms to the STROBE guidelines for human observational studies.

### Oral health examination

Dentists at each dental clinic conducted oral health examinations using detailed manuals to standardize the assessment of oral health status. The condition of each tooth in each participant was recorded as a sound, decayed, filled, or missing. The number of remaining teeth was calculated as the total number of sound, decayed, and filled teeth after excluding third molars. We examined whether prosthetic treatment of missing teeth is necessary as the participant’s need for prosthetic treatment. The community periodontal index (CPI) was used to assess periodontal status^14^ because the method is commonly used in public oral health examinations in Japan. The oral cavity of each participant was divided into six sextants. The index tooth numbers were 11, 16, 17, 26, 27, 31, 36, 37, 46, and 47. Measurements were made at six sites (mesiobuccal, mid-buccal, distobuccal, distolingual, mid-lingual, and mesiolingual) in each index tooth. The CPI scores were coded as follows: healthy (code 0), bleeding after probing (code 1), dental calculus detected by probing (code 2), 4–5 mm shallow pocket (code 3), ≥6 mm deep pocket (code 4), and relevant teeth missing (code X).

### Medical examination

In the medical examination, metabolic components, including cholesterol levels, triglyceride (TG) levels, fasting glucose or glycated hemoglobin (HbA1c) levels, blood pressure, and body mass index (BMI), were assessed. Overnight fasting blood was collected to measure serum blood sugar, TGs, and HDL cholesterol. Height and body weight were measured to calculate the BMI of each participant.

The harmonizing criteria, defined in a joint interim statement of the International Diabetes Federation Task Force on Epidemiology and Prevention; National Heart, Lung, and Blood Institute; American Heart Association; World Heart Federation; International Atherosclerosis Society; and International Association for the Study of Obesity, were used to characterize MetS.^15^ We used these criteria, with the exception of central obesity, because we did not obtain waist circumference data. Instead, we substituted BMI for waist circumference. Participants who met three or more of the following five criteria were defined as having MetS: central obesity (BMI ≥25 kg/m^2^), hypertriglyceridemia (TG level ≥150 mg/dL), low HDL cholesterol levels (<40 mg/dL for men, <50 mg/dL for women), high blood pressure (systolic ≥130 mm Hg and/or diastolic ≥85 mm Hg), and hyperglycemia (fasting plasma glucose level ≥100 mg/dL or HbA1c levels ≥5.6%). Participants using antihypertensive medication or antihyperglycemic medication were defined as having high blood pressure or hyperglycemia, respectively. The diagnostic criteria for hypertriglyceridemia and low HDL cholesterol level to define MetS were based only on biochemical data. Because the data on medication for hyperlipidemia were obtained from the question “Do you take a drug to reduce cholesterol?”, it could not be applied to the criteria hypertriglyceridemia or low HDL cholesterol level. Therefore, we considered data on medication for hyperlipidemia to be a confounder in the multivariate analyses.

### Questionnaire

Information about the participants’ current and previous medical histories; medications; and lifestyle habits, such as smoking (current or never/past), alcohol consumption (everyday, sometimes, or never), exercise habit (yes or no), weight gain since 20 years of age (yes or no), eating speed (slow, normal, or fast), dental checkup frequency (<1 or ≥1 time/year), and use of secondary oral hygiene products, such as dental floss or interdental brushes (everyday, sometimes, or none); was obtained using self-administered questionnaires. Eating speed was assessed with the question “Do you eat faster than others?” Participants who exercised ≥30 min twice weekly for more than 1 year were classified as having an exercise habit. Weight gain was recorded if ≥10 kg weight had been gained since the participant was 20 years of age.

### Statistical analyses

Data from 2,379 participants were used in the analyses. The number of remaining teeth was divided into three categories (20–28 teeth, 10–19 teeth, or 0–9 teeth). Regarding CPI, we divided participants into four categories (codes 0–2, 3, 4, or X), where code X is a dummy code corresponding to an absence of representative teeth.

Differences in the proportions of categorical variables were evaluated using Pearson’s chi-square test. We performed bivariate and multivariate logistic regression analyses to examine the correlations of age, sex, smoking, alcohol consumption, exercise, weight gain, eating speed, medication for hyperlipidemia, dental checkup frequency, secondary oral hygiene products, CPI, and number of teeth with MetS and each component of MetS (central obesity, hypertriglyceridemia, low HDL cholesterol level, high blood pressure, and hyperglycemia). Both unadjusted and covariate-adjusted odds ratios (ORs) and confidence intervals (CIs) were calculated for MetS. Variables that were statistically significant in bivariate analysis were input into multivariate logistic regression analysis. Age, sex, smoking habit, CPI, and number of teeth were entered forcibly into the multivariate analysis, even when they did not show statistical significance in bivariate analyses. In addition, we analyzed the correlations of the variables with MetS separately in men and women to show differences by sex, as previous studies reported sex differences in the relationships of the number of teeth and periodontal status with MetS.^16^^,^^17^ We constructed a variable combining the number of teeth and eating speed and examined the correlation of the combined variable with MetS. All statistical analyses were performed using SPSS ver. 24.0 (IBM Corp., Armonk, NY, USA). A *P*-value <0.05 was considered significant.

## RESULTS

Table 1 shows the characteristics of the participants according to sex. The number of teeth, periodontal status, smoking habit, alcohol consumption, exercise habit, weight gain since 20 years of age, eating speed, and frequency of using secondary oral hygiene products were significantly different between men and women.

**Table 1.** Characteristics of the study participants according to sex

Variable	Men (*n* = 960)	Women (*n* = 1,419)	*P* value^a^
	*n* (%)		
Age			
75 years old	595 (62.0)	874 (61.6)	0.442
80 years old	365 (38.0)	545 (38.4)	
Smoking habit			
Never/past	879 (91.6)	1,407 (99.2)	<0.001
Current	81 (8.4)	12 (0.8)	
Alcohol consumption			
Never	388 (40.4)	1,202 (84.7)	<0.001
Sometimes	205 (21.4)	171 (12.1)	
Everyday	367 (38.2)	46 (3.2)	
Exercise habit			
No	392 (40.8)	723 (51.0)	<0.001
Yes	568 (59.2)	696 (49.0)	
Weight gain since 20 years of age			
No	717 (74.7)	1,135 (80.0)	0.001
Yes	243 (25.3)	284 (20.0)	
Eating speed			
Slow	95 (9.9)	142 (10.0)	0.037
Normal	674 (70.2)	1,025 (74.1)	
Fast	191 (19.9)	225 (15.9)	
Dental checkup frequency			
<1 time/year	456 (47.5)	639 (45.0)	0.127
≥1 time/year	504 (52.5)	780 (55.0)	
Secondary oral hygiene products			
None	492 (51.3)	569 (40.1)	<0.001
Sometimes	213 (22.2)	359 (25.3)	
Everyday	255 (26.6)	491 (34.6)	
Community periodontal index			
Code 0–2	331 (34.5)	541 (38.1)	<0.001
Code 3	345 (35.9)	566 (39.9)	
Code 4	211 (22.0)	203 (14.3)	
Code X	73 (7.6)	109 (7.7)	
Number of teeth			
20–28 teeth	580 (60.4)	814 (57.4)	0.045
10–19 teeth	234 (24.4)	333 (23.5)	
0–9 teeth	146 (15.2)	272 (19.2)	
Need prosthetic treatment			
No	816 (85.0)	1,220 (86.0)	0.272
Yes	144 (15.0)	199 (14.0)	

^a^Pearson’s chi-square test.

Table 2 shows the correlations of oral health, lifestyle factors, and other variables with MetS in both sexes using bivariate and multivariate logistic regression analyses. The prevalence of MetS was 24.0% in men and 23.5% in women. In the bivariate analyses, the number of teeth, exercise habit, weight gain since 20 years of age, eating speed, frequency of using secondary oral hygiene products, and medication for hyperlipidemia were significantly associated with MetS. In the multivariate model, the number of teeth was inversely associated with MetS. Compared to those with 20–28 teeth, participants with fewer teeth had significantly higher ORs for having MetS, and the OR for MetS was highest in the participants with 0–9 teeth. The OR for MetS was significantly higher for participants with a fast eating speed and was significantly lower for participants using secondary oral hygiene products every day. The analysis excluding edentulous elderly persons showed a similar tendency: participants using secondary oral hygiene products daily and had a significantly lower OR for MetS. There were no significant correlations between the number of teeth and each component of MetS, but eating speed was correlated with obesity and hypertriglyceridemia. The OR of eating fast for obesity was 2.92 (95% CI, 1.73–4.95) and that for hypertriglyceridemia was 1.78 (95% CI, 1.16–2.74). Using secondary oral hygiene products every day was inversely correlated with hypertriglyceridemia, and the OR was 0.73 (95% CI, 0.57–0.96).

**Table 2.** Relation of oral health, life-style factors, and other variables to Metabolic syndrome

Variable	Metabolic syndrome		Crude OR (95% CI)	Adjusted OR (95% CI)

	Negative (*n* = 1,816)	Positive (*n* = 563)		
	*n* (%)			
Age				
75 years old	1,129 (62.2)	340 (60.4)	1	1
80 years old	687 (37.8)	223 (39.6)	1.08 (0.89–1.31)	0.99 (0.80–1.22)
Sex				
Men	730 (40.2)	230 (40.9)	1	1
Women	1,086 (59.8)	333 (59.1)	0.97 (0.80–1.18)	0.98 (0.78–1.23)
Smoking habit				
Never/past	1,749 (96.3)	537 (95.4)	1	1
Current	67 (3.7)	26 (4.6)	1.26 (0.80–2.01)	1.28 (0.77–2.14)
Alcohol consumption				
Never	1,215 (66.9)	375 (66.6)	1	—
Sometimes	282 (15.5)	94 (16.7)	1.08 (0.83–1.40)	
Everyday	319 (17.6)	94 (16.7)	0.96 (0.74–1.24)	
Exercise habit				
No	827 (45.5)	288 (51.2)	1	1
Yes	989 (54.5)	275 (48.8)	0.80 (0.66–0.97)	0.82 (0.67–1.01)
Weight gain since 20 years of age				
No	1,559 (85.8)	293 (52.0)	1	1
Yes	257 (14.2)	270 (48.0)	5.59 (4.52–6.91)	5.30 (4.26–6.59)
Eating speed				
Slow	196 (10.8)	41 (7.3)	1	1
Normal	1,343 (77.8)	383 (68.0)	1.36 (0.96–1.95)	1.34 (0.92–1.95)
Fast	277 (15.3)	139 (24.7)	2.40 (1.62–3.56)	2.06 (1.35–3.16)
Intake drug to reduce cholesterol				
No	1,300 (71.6)	332 (59.0)	1	1
Yes	516 (28.4)	231 (41.0)	1.75 (1.44–2.13)	1.62 (1.30–2.01)
Dental checkup frequency				
<1 time/year	826 (45.5)	269 (47.8)	1	—
≥1 time/year	990 (54.5)	294 (52.2)	0.91 (0.76–1.10)	
Secondary oral hygiene products				
None	788 (43.4)	273 (48.5)	1	1
Sometimes	416 (22.9)	156 (27.7)	1.08 (0.86–1.36)	1.19 (0.92–1.54)
Everyday	612 (33.7)	134 (23.8)	0.63 (0.50–0.80)	0.71 (0.55–0.92)
Community periodontal index				
Code 0–2	677 (37.3)	195 (34.6)	1	1
Code 3	700 (38.5)	211 (37.5)	1.05 (0.84–1.31)	1.00 (0.79–1.27)
Code 4	309 (17.0)	105 (18.7)	1.18 (0.90–1.55)	1.18 (0.88–1.60)
Code X	130 (7.2)	52 (9.2)	1.39 (0.97–1.99)	1.10 (0.69–1.77)
Number of teeth				
20–28 teeth	1,092 (78.3)	302 (53.6)	1	1
10–19 teeth	423 (23.3)	144 (25.6)	1.23 (0.98–1.55)	1.23 (0.96–1.58)
0–9 teeth	301 (16.6)	117 (20.8)	1.41 (1.10–1.80)	1.54 (1.10–2.17)
Need of prosthetic treatment				
No	1,553 (85.5)	483 (85.8)	1	—
Yes	263 (14.5)	80 (14.2)	0.98 (0.75–1.28)	

CI, confidence interval; OR, odds ratio.

Table 3 and Table 4 show the correlations between the variables and MetS in men and women, respectively. Having 10–19 teeth and eating fast were positively correlated and using secondary oral hygiene products every day was negatively correlated with MetS in men. Significant correlations between MetS and having 0–9 teeth and eating fast were observed in women.

**Table 3.** Relation of oral health, life-style factors, and other variables to Metabolic syndrome in men

Variable	Metabolic syndrome		Crude OR (95% CI)	Adjusted OR (95% CI)

	Negative (*n* = 730)	Positive (*n* = 230)		
	*n* (%)			
Age				
75 years old	452 (61.9)	143 (62.2)	1	1
80 years old	278 (38.1)	87 (37.8)	0.99 (0.73–1.34)	1.08 (0.77–1.53)
Smoking habits				
Never/past	671 (91.9)	208 (90.4)	1	1
Current	59 (8.1)	22 (9.6)	1.20 (0.72–2.01)	1.28 (0.73–2.25)
Alcohol consumption				
Never	291 (39.9)	97 (42.2)	1	—
Sometimes	156 (21.4)	49 (21.3)	0.94 (0.64–1.40)	
Everyday	283 (38.8)	84 (36.5)	0.89 (0.64–1.25)	
Exercise habit				
No	283 (38.8)	109 (47.4)	1	1
Yes	447 (61.2)	121 (52.6)	0.70 (0.52–0.95)	0.66 (0.48–0.92)
Weight gain since 20 years of age				
No	605 (82.9)	112 (48.7)	1	1
Yes	125 (17.1)	118 (51.3)	5.10 (3.69–7.04)	4.80 (3.43–6.71)
Eating speed				
Slow	75 (10.3)	20 (8.7)	1	1
Normal	530 (72.6)	144 (62.6)	1.02 (0.60–1.73)	0.99 (0.56–1.74)
Fast	125 (17.1)	66 (28.7)	1.98 (1.11–3.52)	1.95 (1.05–3.64)
Intake drug to reduce cholesterol				
No	603 (82.6)	164 (71.3)	1	1
Yes	127 (17.4)	66 (28.7)	1.91 (1.36–2.70)	1.83 (1.24–2.68)
Dental checkup frequency				
<1 time/year	343 (47.0)	113 (49.1)	1	—
≥1 time/year	387 (53.0)	117 (50.9)	0.92 (0.68–1.23)	
Secondary oral hygiene products				
None	366 (50.1)	126 (54.8)	1	1
Sometimes	152 (20.8)	61 (26.5)	1.17 (0.81–1.67)	1.24 (0.83–1.86)
Use everyday	212 (29.0)	43 (18.7)	0.59 (0.40–0.87)	0.59 (0.39–0.90)
Community periodontal index				
Code 0–2	263 (36.0)	68 (29.6)	1	1
Code 3	262 (35.9)	83 (36.1)	1.23 (0.85–1.76)	1.06 (0.71–1.57)
Code 4	153 (21.0)	58 (25.2)	1.47 (0.98–2.19)	1.41 (0.91–2.20)
Code X	52 (7.1)	21 (9.1)	1.56 (0.88–2.77)	1.47 (0.64–3.35)
Number of teeth				
20–28 teeth	460 (63.0)	120 (52.2)	1	1
10–19 teeth	161 (22.1)	73 (31.7)	1.74 (1.24–2.45)	1.75 (1.20–2.55)
0–9 teeth	109 (14.9)	37 (16.1)	1.30 (0.85–1.99)	1.11 (0.59–2.09)
Need of prosthetic treatment				
No	623 (85.3)	193 (83.9)	1	—
Yes	107 (14.7)	37 (16.1)	1.12 (0.74–1.68)	

CI, confidence interval; OR, odds ratio.

**Table 4.** Relation of oral health, life-style factors, and other variables to Metabolic syndrome in women

Variable	Metabolic syndrome		Crude OR (95% CI)	Adjusted OR (95% CI)

	Negative (*n* = 1,086)	Positive (*n* = 333)		
	*n* (%)			
Age				
75 years old	677 (62.3)	197 (59.2)	1	1
80 years old	409 (37.7)	136 (40.8)	1.14 (0.89–1.47)	0.90 (0.68–1.18)
Smoking habit				
Never/past	1,078 (99.3)	329 (98.8)	1	1
Current	8 (0.7)	4 (1.2)	1.64 (0.49–5.48)	1.50 (0.89–5.83)
Alcohol consumption				
Never	924 (85.1)	278 (83.5)	1	—
Sometimes	126 (11.6)	45 (13.5)	1.19 (0.82–1.71)	
Everyday	36 (3.3)	10 (3.0)	0.92 (0.45–1.88)	
Exercise habit				
No	544 (50.1)	179 (53.8)	1	—
Yes	542 (49.9)	154 (46.2)	0.86 (0.68–1.10)	
Weight gain since 20 years of age				
No	954 (87.8)	181 (54.4)	1	1
Yes	132 (12.2)	152 (45.6)	6.07 (4.58–8.05)	5.91 (4.42–7.91)
Eating speed				
Slow	121 (11.1)	21 (6.3)	1	1
Normal	813 (74.9)	239 (71.8)	1.69 (1.04–2.75)	1.64 (0.97–2.75)
Fast	152 (14.0)	73 (21.9)	2.77 (1.61–4.75)	2.18 (1.21–3.95)
Intake drug to reduce cholesterol				
No	697 (64.2)	168 (50.5)	1	1
Yes	389 (35.8)	165 (49.5)	1.76 (1.37–2.26)	1.54 (1.17–2.02)
Dental checkup frequency				
<1 time/year	483 (44.5)	156 (46.8)	1	—
≥1 time/year	603 (55.5)	177 (53.2)	0.91 (0.71–1.16)	
Secondary oral hygiene product				
None	422 (38.9)	147 (44.1)	1	1
Sometimes	264 (24.3)	95 (28.5)	1.03 (0.77–1.40)	1.17 (0.83–1.65)
Use everyday	400 (36.8)	91 (27.3)	0.65 (0.49–0.88)	0.79 (0.57–1.10)
Community periodontal index				
Code 0–2	414 (38.1)	127 (38.1)	1	1
Code 3	438 (40.3)	128 (38.4)	0.95 (0.72–1.26)	0.97 (0.71–1.31)
Code 4	156 (14.4)	47 (14.1)	0.98 (0.67–1.44)	1.00 (0.65–1.51)
Code X	78 (7.2)	31 (9.3)	1.30 (0.82–2.06)	1.00 (0.56–1.81)
Number of teeth				
20–28 teeth	632 (58.2)	182 (54.7)	1	1
10–19 teeth	262 (24.1)	71 (21.3)	0.94 (0.69–1.28)	0.93 (0.66–1.30)
0–9 teeth	192 (17.7)	80 (24.0)	1.45 (1.06–1.97)	1.76 (1.16–2.68)
Need of prosthetic treatment				
No	930 (85.6)	290 (87.1)	1	—
Yes	156 (14.4)	43 (12.9)	0.88 (0.62–1.27)	

CI, confidence interval; OR, odds ratio.

Table 5 shows the correlations of the variable combining the number of teeth and eating speed with MetS. The categories of 0–9 teeth and eating quickly, 10–19 teeth and eating quickly, and 0–9 teeth and eating at normal speed had significantly higher OR for MetS compared with the category of 20–28 teeth and eating slowly.

**Table 5.** Relationship of the variable combining the number of teeth and eating speed with metabolic syndrome

	Eating speed											

	Slow				Normal				Fast			
		
	Negative	Positive	Crude OR (95% CI)	Adjusted^*^ OR (95% CI)	Negative	Positive	Crude OR (95% CI)	Adjusted^*^ OR (95% CI)	Negative	Positive	Crude OR (95% CI)	Adjusted^*^ OR (95% CI)
Number of teeth												
20–28 teeth	97 (5.3)	22 (3.9)	1	1	806 (44.4)	201 (35.7)	1.10 (0.68–1.79)	1.17 (0.70–1.98)	189 (10.4)	79 (14.0)	1.84 (1.08–3.24)	1.64 (0.93–2.91)
10–19 teeth	49 (2.7)	7 (1.2)	0.63 (0.25–1.58)	0.91 (0.35–2.39)	314 (17.3)	95 (16.9)	1.33 (0.80–2.24)	1.34 (0.77–2.34)	60 (3.3)	42 (7.5)	3.09 (1.68–5.67)	2.83 (1.46–5.50)
0–9 teeth	50 (2.8)	12 (2.1)	1.06 (0.48–2.31)	1.16 (0.49–2.76)	223 (12.3)	87 (15.5)	1.72 (1.02–2.91)	1.85 (1.01–3.38)	28 (1.5)	18 (3.2)	2.83 (1.34–6.01)	2.48 (1.06–5.78)

CI, confidence interval; OR, odds ratio.

^*^Adjusted for age, sex, smoking habit, exercise habit, weight gain since 20 years of age, intake drug to reduce cholesterol, using secondary oral hygiene products, and Community periodontal index.

## DISCUSSION

This study showed that of oral health measurements, only the number of teeth that remained was significantly associated with MetS, and periodontal condition was not related to MetS in this aged population. Concerning the lifestyle factors, eating speed and frequency of using secondary oral hygiene products were correlated with MetS, and there was no correlation between MetS and other factors, such as smoking and exercise.

Poor dentition decreases chewing ability^7^ and affects food selection and nutrition intake.^18^ The loss of several teeth is associated with a higher intake of carbohydrates,^19^ and a higher intake of carbohydrates was associated with MetS.^20^ Therefore, the increase in carbohydrate intake caused by tooth loss is thought to be responsible for MetS. Consuming fruits and vegetables reduces the risk of CVD,^21^ whereas loss of several teeth decreases intake of protein; vitamins C, E, and B_6_; and minerals, vegetables, and fruits.^19^ Indeed, tooth loss is associated with the prevalence of, and mortality from, CVD.^22^^,^^23^ As the nutritional imbalance due to losing several teeth can affect systemic health, maintaining as many teeth as possible could potentially prevent MetS.

This study examined the influence of losing several teeth on having MetS. By contrast, other studies have shown that MetS increases the risk of tooth loss.^5^^,^^24^ There is an increase in the proportion of individuals with tooth loss due to periodontal disease after the age of 45 years.^25^ As individuals with MetS often have advanced periodontal disease,^26^ periodontal disease could be an important cause of tooth loss in those with MetS. It is conceivable that MetS and tooth loss influence each other reciprocally considering the bidirectional relationship between obesity and tooth loss.^27^

In this study, the highest OR for MetS was observed in the 10–19 teeth category in men and in the 0–9 teeth category in women, and a sex difference in the relationship between the number of teeth and MetS was apparent. One study examined the relationship between tooth loss and MetS in Korean individuals ≥40 years of age; women with few teeth had a significantly higher risk of MetS, but no significant association was observed in men.^17^ Wearing full dentures improves chewing ability and increases the number of chewable foods in edentulous men compared with edentulous women. In addition, masticatory and physical performances are correlated with wearing full dentures in men but not in women.^28^ Although most participants who had lost several teeth used a prosthesis in the present study, the sex difference in the relationship between tooth loss and MetS may be caused by a sex difference in the recovery of masticatory ability by wearing dentures.

This study suggested that eating quickly was correlated with significantly higher odds of MetS compared with eating slowly. A cohort study of 40–75-year-old participants found that eating quickly resulted in a significantly higher risk of MetS.^29^ Eating quickly makes it difficult to feel full and leads to overeating.^30^ In addition, eating quickly also causes insulin resistance,^31^ and increases the risks of obesity and MetS. Individuals eating quickly who had fewer teeth had a higher OR for MetS than those who ate slowly and had many teeth. Eating quickly and tooth loss were independently associated with MetS, and the coexistence of these two factors increased the risk of MetS. Therefore, as eating quickly is believed to be associated significantly with MetS, dental professionals should provide dietary instructions about eating speed, especially in those who have lost many teeth.

In this study, individuals using secondary oral hygiene products, such as dental floss and interdental brushes, every day had a significantly lower OR for MetS. A 3-year cohort study indicated that frequent tooth brushing reduces the risk of MetS.^32^ Frequent tooth brushing is also associated with a lower risk of CVD.^33^ Individuals who brush their teeth frequently also tend not to smoke or drink, to exercise habitually, and to receive regular health checkups.^34^ Therefore, individuals who brush their teeth frequently and use secondary oral hygiene products likely have an interest in general and oral health, and consequently their lifestyles are often healthier. Tooth brushing and use of secondary oral hygiene products prevent oral diseases, and these may act as barometers for the risk of systemic diseases, as oral health behaviors reflect general lifestyle habits.

Many studies have reported a relationship between periodontal disease and MetS,^9^^,^^16^^,^^26^ but we found no significant correlation between the two. Although most previous studies examined the relationship between periodontal disease and MetS in young or middle-aged populations, our study participants were aged individuals, which may be why no correlation was found. Furuta et al reported that the association between periodontal disease and MetS was stronger in women than in men.^16^ A study of postmenopausal women reported no association between periodontal disease and MetS.^35^ The relationship between periodontal disease and MetS is possibly influenced by age and sex. In this study, the CPI was applied to assess periodontal health status, and its use may be another reason for the lack of a correlation between periodontal status and MetS. Although the CPI is a useful and simple method of evaluating periodontal status, it is possible that the severity of periodontal disease was not understood accurately or that deep periodontal pockets were overlooked, since only representative teeth were evaluated. Evaluating periodontal status using an extensive measuring method would be more likely to reveal the relationship between periodontal disease and MetS.

Several limitations to this study should be mentioned. As this study was cross-sectional, it is impossible to determine causal associations or the direction of the relationships between MetS and oral health and lifestyle factors, such as the number of remaining teeth, eating speed, and frequency of using secondary oral hygiene products. Thus, additional prospective studies are needed. Although an advantage of this study was the large number of study participants, the participants were a convenience sample and the response rate for health examinations was low. Therefore, as there was a selection bias regarding the study participants, there is a limit to the representativeness of the results. As the health examination data were collected from a large region by the local government for the elderly, each participant received medical and oral examinations at their closest medical institution. Therefore, some of the medical items were evaluated differently among the various medical institutions. For example, hyperglycemia is assessed using HbA1c in some institutions and using fasting blood glucose levels in other institutions. The definition of MetS somewhat differed from the original diagnostic criteria because BMI was a substitute for waist circumference. We were unable to confirm specific drug names from the questionnaire on medication, so we could not assess medications related to lipid abnormalities when judging MetS. Oral examinations are performed based on detailed manuals, but the oral examination is not standardized using calibration training among dentists. It was insufficient to adjust for socioeconomic confounders, such as education level, household income, and family history. Furthermore, we could not adjust for factors related to diet and intake of nutrients because nutrition surveys were not performed. Because the participants of this study received health examinations voluntarily, they probably had a high interest in their own health and were proactive enough to receive the health checkups by themselves. Therefore, there might be differences in health status among individuals who receive health examinations and those who do not, so it is difficult to generalize the results.

In conclusion, the results of this study suggest that of oral health measurements, such as the number of remaining teeth, eating speed, frequency of using secondary oral hygiene products, and the combination of the number of teeth and eating speed, were associated with MetS in an aged population. Maintaining teeth and oral health guidance are very important for preventing MetS in the aged population. However, because evidence for the relationship between oral health, lifestyle factors, and MetS in the elderly is still insufficient, additional studies are needed to clarify the mechanism and causal relationship between oral health, lifestyle factors, and MetS and to demonstrate that oral health maintenance contributes to MetS prevention.
